# What is the cognitive footprint of insular glioma?

**DOI:** 10.3389/fnhum.2024.1382380

**Published:** 2024-05-27

**Authors:** Noah M. Nichols, Bahie Ezzat, Allison C. Waters, Fedor Panov, Raymund L. Yong, Isabelle M. Germano

**Affiliations:** ^1^Department of Neurosurgery, Mount Sinai School of Medicine, New York, NY, United States; ^2^School of Medicine, Mount Sinai School of Medicine, New York, NY, United States; ^3^Department of Neuroscience, Mount Sinai School of Medicine, New York, NY, United States

**Keywords:** insular glioma, cognition, functional outcomes, connectomics, brain tumor surgery

## Abstract

Cognitive impairment has a profound deleterious impact on long-term outcomes of glioma surgery. The human insula, a deep cortical structure covered by the operculum, plays a role in a wide range of cognitive functions including interceptive thoughts and salience processing. Both low-grade (LGG) and high-grade gliomas (HGG) involve the insula, representing up to 25% of LGG and 10% of HGG. Surgical series from the past 30 years support the role of primary cytoreductive surgery for insular glioma patients; however, reported cognitive outcomes are often limited to speech and language function. The breath of recent neuroscience literature demonstrates that the insula plays a broader role in cognition including interoceptive thoughts and salience processing. This article summarizes the vast functional role of the healthy human insula highlighting how this knowledge can be leveraged to improve the care of patients with insular gliomas.

## Highlights

- The human insula plays a role in a wide range of cognitive functions including interceptive thoughts and salience processing.
- Insular gliomas represent up to 25% of low-grade gliomas and 10% of high-grade gliomas.
- Many large case series over the past 30 years have demonstrated acceptable rates of morbidity and mortality after insular glioma surgery.
- Language outcomes following insular tumor surgery have been widely reported; however, other aspects of higher cognition are often not discussed.
- The breadth of recent neuroscience studies examining the functional role of the human insula have not been fully translated to patients with intrinsic tumors in this region.

## Introduction

Insular gliomas, which comprise roughly 25% of low-grade gliomas (LGG) and 10% of high-grade gliomas (HGG), pose a unique challenge to neurosurgeons given their complex anatomy (Duffau and Capelle, 2004; Renfrow et al., 2023). Cognitive impairment has a profound detrimental impact on long-term outcomes of glioma surgery including quality of life (QoL) and return to work status (Liu et al., 2009; Sanai et al., 2011; Hameed et al., 2018; Noll et al., 2018; Li et al., 2020). Evidence over the past 30 years suggests that insular glioma surgery can be safely performed with an acceptable morbidity profile (Sanai et al., 2011; Wu et al., 2011; Li et al., 2020). However, while most surgical series report perioperative speech and language outcomes, the breadth of recent neuroscience studies examining the functional role of the human insula have not been fully translated to patients with intrinsic tumors in this region. Specifically, there is a paucity of data describing how the vast cognitive functions of the insula are impacted by gliomas and their treatment (Table 1).

**Table 1:** Summary of literature describing functional outcomes in insular glioma patients.

First author	Year	Cognitive domain	Objective	Design	Methods/Task	Number of patients	Low-grade glioma (LGG) %	Main findings
Duffau	2009	Speech/Language	Describe functional outcomes of awake intraoperative language mapping in patients with dominant hemisphere insular glioma	Retrospective case series	Intraoperative counting and picture naming tasks	24	100	Preoperative dysphasia observed in 29%. 5 patients had language positive sites in the insula. All patients recovered to pre-operative language function status, and 6 patients had improvement in pre-operative language dysfunction
Wu	2011	Comprehensive	Characterize pre-and-post operative cognitive function in patients with insular gliomas	Retrospective enrollment of cases and controls	Comprehensive neurocognitive task-based assessment performed pre-and post-op	33	55	Patients with insular tumors had significantly worse preoperative performance on naming tests
Chen	2016	Social	Describe the impact of insular gliomas on cognitive and affective empathic abilities	Retrospective enrollment of cases and controls	Neuropsychological battery of questionnaires	46	39	Lower alexithymia scale scores as well as lower scores on cumulative AE and CE scales in insular glioma patients compared to controls
Zarino	2021	Speech/Language	Characterize language impairment pre-and post-surgery in insular glioma patients	Retrospective analysis of prospectively collected data	Comprehensive language assessment battery	35	26	Language performance worsened in the acute postoperative period. 44% of patients with dominant hemisphere insular glioma and preoperative language deficit had improved language function after surgery but still pathologic
Gomez-Andres	2022	Attention (Salience)	Explore the functional role of the aIC for self- monitoring in a patient undergoing awake craniotomy for tumor resection	Prospective enrollment of glioma patients	Intraoperative Stroop Task with DCS	1	100	Of the 4 total aIC stimulated sites, only 1 (25%) was associated functional disruption of self-monitoring. The was site was located at the posterior limit of the aIC

aIC, Anterior insular cortex; DCS, Direct Cortical Stimulation.

Clinically relevant cognitive impairment is caused by insular glioma as well as surgical resection. The higher-level cognition in patients with LGG and HGG involving the insula was first characterized in 2011. Patients with insular gliomas demonstrated poorer preoperative performance on visual confrontational naming tasks compared to matched controls (Baker et al., 2018). There was also a tendency for decline in the domains of learning, memory, executive function, and motor function (Wu et al., 2011). This study first suggested that cognitive deficits often go undetected outside of the research setting.

In this review article, we aim to sketch the cognitive footprint of insular glioma in a way that provides recommendations for neuropsychological testing and management of patients affected by this disease.

## The function of the insula

Despite tremendous interest from the neuroscience community, the insula remains one of the least understood cerebral regions. In 1955, Penfield and Faulk provided the first clues to insular function when they published findings from direct cortical stimulation (DCS) of the insula after temporal lobectomy in epilepsy patients. Patients reported a spectrum of sensations in contralateral body parts including warmth, numbness, shock, and tightness. Most stimulation sites were in the inferior and posterior insula as the superior region was poorly exposed after temporal lobectomy (Penfield and Faulk, 1955).

**Figure 1:**
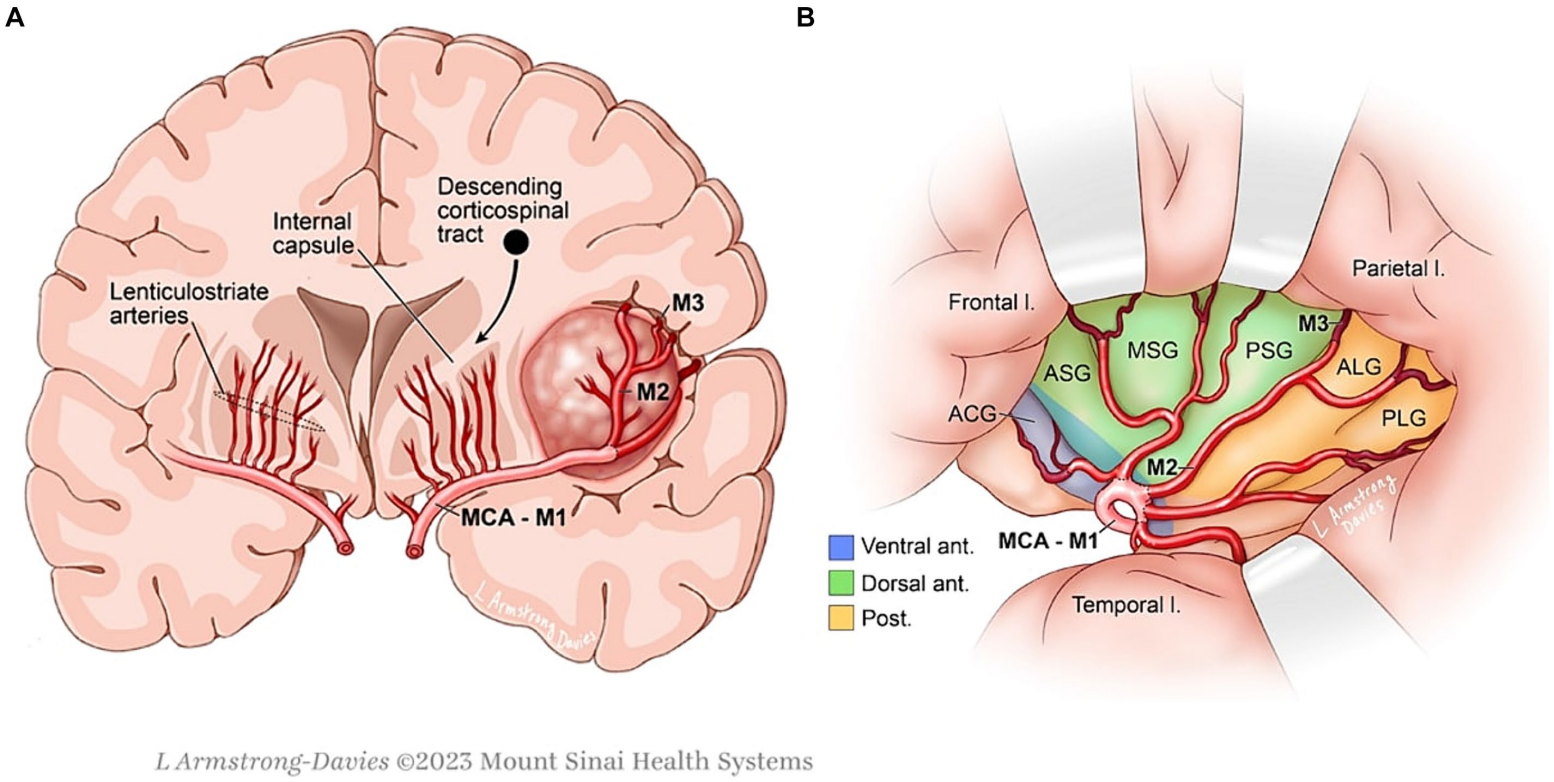
**(A)** Surgical anatomy of the insulo-sylvian region relative to an insular glioma. **(B)** Healthy human insula with associated microvasculature and functional topography.

In more recent years we have come to realize that the complex anatomical features of the insula are matched by an equally as complex functional role (Table 2). The insula has functionally specific spatial organization. A 2010 large meta-analysis of functional neuroimaging experiments revealed at least three distinct functional regions (Kurth et al., 2010). The dorsal anterior insula (dAI) has connections to the frontal lobe, anterior cingulate, and parietal areas, and is most associated with cognition. The ventral anterior insula (vAI) with connections to limbic areas appears to play a role in socio-emotional processing. The posterior insula (PI) is most associated with sensorimotor processing and there is also a central region which is implicated in olfactory and gustatory function (Figure 1) (Kurth et al., 2010). The emergence of the Human Connectome Project (HCP) and connectomics have led to even further parcellations, with currently 13 insular subdivisions (Glasser et al., 2016; Baker et al., 2018; Morell et al., 2022). While the technical aspects of MRI acquisition vary depending (i.e., oncologic purposes compared to stereotactic electrode implantation or studying healthy populations), standard sequences, including diffusion weighted sequences, performed on 1.5 or 3.0 Telsa machines are adequate (Naidich et al., 2004; Uddin et al., 2014; Nomi et al., 2016; Uddin et al., 2017; Blustajn et al., 2019).

**Table 2:** Functions of the human insula.

Category	Function	Laterality	Location
Cognition	Salience	Right	Anterior
	Speech	Left	Anterior
Sensorimotor Processing	Interoception	Right	Anterior
	Pain	Bilateral	Dorsal Posterior
	Auditory	Bilateral	Anterior and Posterior
	Vestibular	Bilateral	Posterior
	Chemosensory	Bilateral	Middle
Socio-emotional Processing	Emotion	Bilateral	Anterior
	Empathy	Bilateral	Anterior

### Speech and language: the unclear but important role of the dominant insula

The role of the insula in speech and language remains controversial. The insula was first implicated in speech function in 1996 when a distinct deficit in articulatory planning was observed in all patients with strokes involving the dominant anterior insula and completely absent in stroke patients with this region spared (Dronkers, 1996). However, by 2019 there was convincing evidence that the insula’s role in speech function was far different. By recording bihemspheric cortical activity with stereo-electroencephalographic (sEEG) electrodes in patients undergoing seizure location, only sparse signaling from frontal operculum and not the insula was recorded immediately before speech output. Instead, activity in the bilateral posterior insula was highest after speech articulation (Woolnough et al., 2019).

The rapid expansion of studies on the neurobiology and mechanisms underlying human speech and language have contributed to a contemporary dual-stream model. The dorsal stream, which includes the superior temporal gyrus, is associated with language processing, spectro-temporal and phonological analysis. The ventral stream, which involves the anterior and middle temporal gyri, facilitates speech recognition and lexical recall (Chang et al., 2015). Recent studies are that the anterior insula plays a role in higher-order cognitive aspects of speech and language processing involving both dorsal and ventral streams (Oh et al., 2014). For example, Cesare et al. demonstrated increased activation on fMRI in the central insula in response to “vitality effects,” which are social cues, like tone or body language, which influence perception of speech (di Cesare et al., 2018).

The specific impact of insular gliomas on language function is not clear, however, several studies have reported mild dysphasia as a presenting symptom in 6–9% of patients with dominant hemisphere insular glioma (Duffau et al., 2009; Wu et al., 2011; Skrap et al., 2012). In a series of 24 patients with dominant hemisphere insular LGG a pre-operative language impairment was noted in of 29% of patients (Chang et al., 2015). Furthermore, 21% of patients had at least one site on the dominant insular cortex where speech arrest was induced with DCS (Chang et al., 2015). The ability to maintain normal language function despite tumor infiltration of the dominant insula may be an example of plasticity.

A more recent study with 35 insular glioma patients who underwent a robust perioperative language assessment showed worse speech performance on all language tasks in patients with dominant hemisphere gliomas {need to add ref.# here]. Patients with fronto-temporo-insular and pure insular gliomas were most likely to present with pre-operative pathologic scores. Language performance worsened in the acute postoperative period. Interestingly, 44% of patients with dominant hemisphere glioma did not recover from aphasic symptoms 3 months after surgery and exhibited a persistent language impairment, albeit improved compared to preoperative and immediate postoperative performance. Moreover, patients with pure dominant hemisphere insular glioma had pathologic scores in the Token Test (TT), Object Naming (ON), Verb Naming (VN), Phonemic Fluency (PF), and Semantic Fluency (SF).

### Interoception: awareness of the internal self affects outward behavior

How do you know that your heart is racing before you give a presentation to a large audience? How do you know that you are full when your stomach becomes distended after a meal? How do these two scenarios affect our behavior? Interoception is the summation of internal stimuli which allows us to answer the following question – How do you feel? This is accomplished by ascending viscerosensory inputs that arrive to the insula via the thalamus where they are processed (Craig, 2002; Mazzola et al., 2009; Critchley and Harrison, 2013; Tayah et al., 2013). Neuroimaging studies have demonstrated insular activation in response to non-painful tactile as well as painful tactile stimulation (zu Eulenburg et al., 2013). By analyzing changes in whole-brain cerebral blood flow (CBF), a strong correlation between increased CBF in the insula and pain scores were noted (Baier et al., 2014). Additionally, the authors overlaid cluster data and coordinates from previously published studies which suggests somatotopic organization within the insula as well (Segerdahl et al., 2015). These findings support a central role of the insula in pain perception which has been further demonstrated in other studies (Craig et al., 2000; Baier et al., 2014; Jensen et al., 2016). Currently, interoceptive processing is thought to progress in a posterior to anterior direction in which interoceptive stimuli are received by the posterior insula and integrated into perceptual maps in the anterior insula (Craig, 2009; Oh et al., 2014).

Interoceptive processing affects our outward behavior. Emotions are among the core aspects of human awareness and James-Lange first proposed that emotions are first activated by bodily changes (Northoff, 2012). Several functional imaging studies have demonstrated activation of the anterior insula in association with negative emotional experiences, positive emotional experiences, disgust, and even sexual pictures (Pugnaghi et al., 2011; Boucher et al., 2015; Papagno et al., 2016). Similarly, the ability to perceive the emotions and empathy of others also appears to activate the insula. Empathy is a neurocognitive behavioral construct that requires sharing of an emotion which is matched by an appropriate/reciprocal inference of feelings, motivations, and subsequent behavior. Affective empathy (AE) is the ability to experience an appropriate empathetic response to another’s emotional state while cognitive empathy (CE) is the capacity to predict and understand another’s mental state using cognitive processes. Specifically, the left insular cortex is associated with affective as well as cognitive forms of empathy, while the right insular cortex only demonstrated an association with the affective form (Fan et al., 2011).

Interoception and emotional dysfunction have not been adequately characterized in patients with insular glioma. Perhaps the most rigorous examination of this topic was done by Chen et al. in which the authors investigated the impact of insular glioma on AE and CE. They reported lower alexithymia scale scores as well as lower scores on cumulative AE and CE scales in insular glioma patients compared to controls (Chen et al., 2016). There were no differences in outcomes based on tumor laterality. While novel and informative, this study did not address how tumor volume, location, and degree of insular involvement impact outcomes.

### Salience processing – which stimuli deserve our attention?

**Figure 2:**
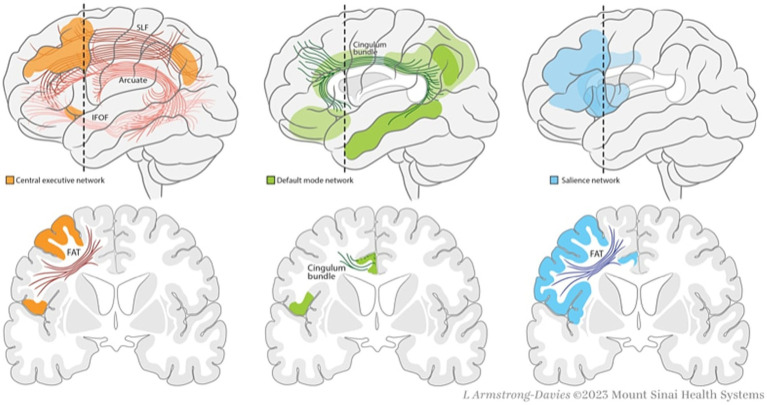
Non-Traditional Eloquent Networks (NTENs) and associated white matter tracts. The central executive network (CEN) consists of hubs in posterior parietal cortex (PPC) and dorsolateral prefrontal cortex (DLPFC). The arcuate fasciculus (AF), superior longitudinal fasciculus (SLF), inferior fronto-occipital fasciculus (IFOF), and frontal aslant tract (FAT) are the major associate bundles of the CEN. The default mode network (DMN) consists of nodes in anterior and posterior cingulate, lateral parietal cortex, precuneus, ventral medial prefrontal cortex (VMPFC), and anterolateral middle temporal cortex. The cingulum bundle and U fibers provide the majority of white matter connections between DMN hubs. The salience network (SN) consists of hubs in the anterior insula, frontal operculum, dorsal prefrontal cortex, and anterior cingulate cortex. The FAT is the major white matter tract of the SN.

We are constantly bombarded with multiple internal and external stimuli and yet not all occupy our attention to the same degree. Instead, we are able to discern which stimuli are “salient” or important. Salience processing is not always a conscious mental task as it also incorporates visceral and autonomic stimuli (Seeley et al., 2007). Our ability to have focused goal-directed behavior relies on intact salience processing. Connectomics has demonstrated that salience processing is not found in a single cerebral region but rather diffusely localized to several hubs, or networks, which demonstrate coordinated activity during neurocognitive tasks. The insula, specifically the dAI, has been intricately associated with Salience Network (SN) (Uddin, 2015). The SN also includes hubs in the frontal operculum, dorsal prefrontal cortex, and anterior cingulate cortex (Schimmelpfennig et al., 2023). The frontal aslant tract (FAT) provides nearly half of the connections between SN nodes while the remaining connections are supplied by association U-fibers (Figure 2) (Papagno et al., 2016; la Corte et al., 2021; Briggs et al., 2022).

Salience processing is enacted by a regulatory effect on other key networks, the Default Mode Network (DMN) and the Central Executive Network (CEN). The DMN demonstrates decreased activity during attention demanding tasks and increased activity during introspective thought and socially cognitive tasks (Frith and Frith, 1999; Raichle et al., 2001; Kelley et al., 2002). Reciprocally, the CEN demonstrates increased activity during attention-demanding tasks. There is emerging evidence which suggests that our ability to execute goal-directed, cognitively demanding tasks involves coordinated regulation of the DMN and CEN. In this “Triple Network Model” the SN is able to influence behavior by detecting salient stimuli, upregulating activity in the CEN, and downregulating activity of the DMN (Sridharan et al., 2008; Menon and Uddin, 2010). SN, DMN, and CEN have been coined non-traditional eloquent networks (NTENs) because of their newly recognized importance compared to well-described peri-sylvian language pathways and motor pathways. Insular activation has been demonstrated with several salience-related tasks such as the Stroop task, stop-signal task, and Simon task (Sharp et al., 2010; Ham et al., 2013; Gomez-Andres et al., 2022).

Several studies have examined NTEN integrity in glioma patients; however, the overarching theme is that while these networks are often affected by gliomas, the correlation with cognitive outcomes has largely gone unexplored. A recent study reported on 85 patients with insular gliomas and compared topologic changes in gray matter and fractional anisotropy in SN hubs between insular glioma patients and healthy controls (Hu et al., 2023). They demonstrated that insular glioma patients had decreases in FA and gray matter in the insula, basal ganglia, and ACC compared to healthy controls, but neurocognitive outcomes were not reported. A cloud-based machine learning platform to evaluate large-scale networks in brain tumor patients was reported by two investigators (Uddin et al., 2014; Hu et al., 2023). NTENs were affected in 93% of patients in one study, however this study did not include insular glioma patients (Uddin et al., 2014). The other study only enrolled insular glioma patients and the SN was affected in 60% of that cohort, but, again, these findings were not translated to cognitive outcomes (Wu et al., 2023).

## Insular glioma surgery today

Insular gliomas were historically considered inoperable lesions given the surrounding complex anatomy. The insula is located at the depths of the sylvian fissure and is covered by the opercula of the frontal, parietal, and temporal lobes which are eloquent on the dominant side. It is anatomically segmented by three anteriorly projecting short gyri (posterior, middle, and anterior) and two posteriorly projecting long gyri (anterior and posterior) (Tanriover et al., 2004). The middle cerebral artery, with its perforator branches from the M2 segments, along with lenticulostriate arteries, provides the majority of the blood supply to the insula. These vascular structures often pose the most significant risks in insular glioma surgery and the lenticulostriate arteries in particular limit the medial extent of resection. Additionally, the insula is surrounded by eloquent white matter tracts: arcuate fasciculus (AF), superior longitudinal fasciculus (SLF), uncinate fasciculus (UF), inferior fronto-occipital fasciculus (IFOF), and the corticospinal tract (CST) (Dziedzic et al., 2022).

Yaşargil et al. (1992) was the first to describe the transsylvian approach to insular tumors which he categorized based on location. The Berger-Sanai Classification has become the most common classification and can be used to predict extent of resection (EOR) and aid surgical planning. In this location-based classification, the insula is divided into zones (I anterior-superior; II posterior-superior; III posterior-inferior; and IV anterior-inferior) using a line along the sylvian fissure and bisected by a perpendicular line through the foramen of Monroe (Wu et al., 2011). Applying modern surgical techniques has extended the range of surgical options, leading to targeted resections with acceptable morbidity profiles, such as permanent speech deficits in 0–5% of patients and motor deficits in 2–10%.

There has been a shift in operative approach over the past 20 years. The transcortical method through non-functional “windows” is a common technique given the increased risk of retraction-induced ischemic damage from the transsylvian approach (Duffau et al., 2006; Menon and Uddin, 2010; Wu et al., 2011; Przybylowski et al., 2019; Pitskhelauri et al., 2021). The existing literature suggests that the morbidity profile for modern insular glioma surgery is acceptable (Table 3). While new or worsening language and motor deficits immediately after surgery are common, these symptoms are often transient with the majority of patients having resolution several months after surgery (Kurth et al., 2010; Wu et al., 2011; Ham et al., 2013; Glasser et al., 2016). Permanent speech and motor deficits are observed in 0–5% and 2–10%, respectively. It is important to note, however, that a new language deficit particularly speech, is associated with worse overall survival (OS). Speech deficits are commonly expressive in nature; however, with such limited data describing the effect of insular resection on NTENs, additional studies are needed. While preserving speech and motor function has been a central tenant of neurosurgical oncology, with respect to insular glioma, these neurologic functions merely scratch the surface of the functional capacity of the human insula. In fact, there is evidence that the human insula plays vital role in cognition and behavior, but the clinical impact of intrinsic neoplasms in this critical cerebral region remains poorly understood.

**Table 3:** Selected surgical series for insular glioma patients.

First author	Year	Number of patients *N* (%)	Low-grade glioma *N* (%)	Most common type^a^	Dominant hemisphere *N* (%)	Percent transcortical	Percent awake craniotomy	Percentage of transient speech/motor deficits		Percentage of permanent speech/motor deficits	
Yaşargil	1992	177	100 (56)	Type 5A	94 (53.1)	0	NS	NS	NS	5^b^	
Duffau	2000	12	12 (100)^d^	Type 5A	2 (16.6)	100	25	8	50	0	8
Lang	2000	22	11 (50)	NS	13 (59)	36	23	27	18	0	9
Duffau	2006	42	42 (100)^d^	Type 5A	12 (28.6)	100	29	23	50	0	7
Duffau	2009	51	51 (100)^d^	Type 5A	14 (27.5)	94	31	19	37	0	3.9
Simon	2009	94	36 (38)	Type 5A	38 (40.4)	75^e^	0	NS	NS	13	13
Sanai	2010	104	70 (60)	Zone I	55 (55.8)	100	57	4.5	7.7	0	1.9
Skrap	2011	66	53 (80)^c^	Type 5A	44 (66)	NS	65	11	11	2	2
Hervey-Jumper	2016	114	62 (54)	Zone I	60 (52.7)	100	45	16	19	0.8	2.6
Hameed	2019	255	201 (78)	Zone I	145 (56.8)	100	NS	5.5	3.5	4.7	8.2
Przybylowski	2020	100	32 (32)	Zone III	60 (60)	48	12	5.0	13.0	0.0	10
Li	2020	253	149 (58.9)	Zone I	119 (47.0)	100	0	9	11	1	2
Pitskhelauri	2021	79	53 (67)	Giant	49 (62)	0	3	16	10	1	5

a Reported as Berger-Sanai or Yaşargil classification.

b Combined rate of permanent speech and motor deficits.

c Study only included non-enhancing lesions.

d Selected only low-grade glioma.

e Surgical technique included lobectomies for exposure.

NS, Not Specified.

## Future directions

The steady flow of new studies describing the function of the insula has outpaced our ability to translate this knowledge towards management of insular glioma patients. Despite the complex functional role of the insula, many patients with insular glioma present without a noticeable clinical deficit. It is possible, however, that if they were examined by neuro-psychological testing, the presence of cognitive deficits could be identified. Addressing this problem is challenging because of obstacles to widespread and routine cognitive testing for glioma patients. Additionally, patients might have poor insight to their degree of cognitive impairment (Tucha et al., 2000). Functional plasticity is a phenomenon that likely explains these findings (Sanai et al., 2008; Satoer et al., 2016). An example is absence of apparent language impairment in dominant hemisphere of insular glioma patients despite tumor invasion of the operculum. Plasticity however often does not fully preserve function (Berzero et al., 2021).

The potential for radiographic biomarkers of cognitive function in brain tumor patients is emerging. Tumor volume was associated with effect on preoperative cognitive function in patients with LGG, including insula, as quantified by standard MRI sequences and voxel-based lesion symptom mapping (VLSM) The study consisted of 8 patients with dominant hemisphere insular glioma, and they demonstrated significantly poor performance in object naming. Additionally, VLSM revealed that for both left and right hemispheric glioma groups, 36% of patients with working memory (verbal and visual) impairment had insular involvement (Guarracino et al., 2022).

In a similar study using resting state fMRI, ipsilateral and contralateral connectivity in treatment-naive patients with insular LGG were examined and demonstrated the functional plasticity specifically exhibited by the dominant insula. The authors report significantly increased functional connectivity in 33 edges originating from the dominant insular lobe in patients with non-dominant insular gliomas. This suggests that when glioma cells infiltrate the non-dominant insula, the dominant hemisphere compensates by strengthening original functional connections. In contrast, when LGG originates in the dominant insular lobe, the non-dominant insular lobe must compensate by increasing connectivity to nodes in bilateral hemispheres (Fang et al., 2021). Identifying the biologic factors that alter network dynamics will help to define the cognitive impact of insular glioma.

## Conclusion

Understanding of cognitive outcomes in insular glioma patients has evolved yet remains incomplete. The insula’s function as a hub for multimodal sensory processing and emotional regulation further complicates the assessment of these deficits. Modern surgical techniques have decreased the rate of permanent speech and motor deficits. Despite these advances, a significant gap persists in understanding the long-term cognitive and subclinical impacts on patients, especially as survival rates improve. Promising avenues for future research include the use of advanced neuroimaging to characterize cognition radiographically and studies aimed at elucidating the biological factors that alter network dynamics. As the focus shifts toward supramaximal safe resections, further exploration is warranted in three specific areas: 1) the complex functions of the insula to better predict cognitive outcomes, 2) the impact of surgical techniques on cognitive functions, and 3) the development of reliable methods for longitudinal cognitive assessment. Such comprehensive research will allow us to have a meaningful impact on the quality of life for insular glioma patients by understanding with greater clarity the cognitive burden of their disease and risk–benefit profile of surgical intervention.

## Author contributions

NN: Conceptualization, Data curation, Formal analysis, Investigation, Methodology, Project administration, Resources, Software, Supervision, Validation, Visualization, Writing – original draft, Writing – review & editing. BE: Conceptualization, Data curation, Formal analysis, Investigation, Writing – original draft, Writing – review & editing. AW: Conceptualization, Investigation, Methodology, Writing – original draft, Writing – review & editing. FP: Conceptualization, Investigation, Methodology, Supervision, Writing – original draft, Writing – review & editing. RY: Conceptualization, Investigation, Supervision, Writing – original draft, Writing – review & editing. IG: Conceptualization, Investigation, Methodology, Project administration, Supervision, Validation, Visualization, Writing – original draft, Writing – review & editing.
